# Concordance of real-world versus conventional progression-free survival from a phase 3 trial of endocrine therapy as first-line treatment for metastatic breast cancer

**DOI:** 10.1371/journal.pone.0227256

**Published:** 2020-04-21

**Authors:** Cynthia Huang Bartlett, Jack Mardekian, Matthew James Cotter, Xin Huang, Zhe Zhang, Christina M. Parrinello, Ariel Bulua Bourla

**Affiliations:** 1 Pfizer Inc, New York, NY, United States of America; 2 Flatiron Health, New York, NY, United States of America; University of Nebraska Medical Center, UNITED STATES

## Abstract

There is growing interest in leveraging real-world data to complement knowledge gained from randomized clinical trials and inform the design of prospective randomized studies in oncology. The present study compared clinical outcomes in women with metastatic breast cancer who received letrozole as first-line monotherapy in oncology practices across the United States versus patients in the letrozole-alone cohort of the PALOMA-2 phase 3 trial. The real-world cohort (N = 107) was derived from de-identified patient data from the Flatiron Health electronic health record database. The clinical trial cohort (N = 222) comprised postmenopausal women in the letrozole-alone arm of PALOMA-2. Patients in the real-world cohort received letrozole monotherapy per labeling and clinical judgment; patients in PALOMA-2 received letrozole 2.5 mg/d, continuous. Real-world survival and response rates were based on evidence of disease burden curated from clinician notes, radiologic reports, and pathology reports available in the electronic health record. Progression-free survival and objective response rate in PALOMA-2 were based on Response Evaluation Criteria in Solid Tumors v1.1. Concordance of survival and response rates were retrospectively assessed using inverse probability of treatment weighting-adjusted Cox regression analysis. Inverse probability of treatment weighting-adjusted Cox regression results showed similar median progression-free survival in the real-world and PALOMA-2 cohorts (18.4 and 16.6 months, respectively): the hazard ratio using real-world data as reference was 1.04 (95% CI, 0.69–1.56). No significant difference was observed in response rates: 41.8% in the real-world cohort vs 39.4% in the PALOMA-2 cohort (odds ratio using real-world data as reference: 0.91 [95% CI, 0.57–1.44]). These findings indicate that data abstracted from electronic health records with proper quality controls can yield meaningful information on clinical outcomes. These data increase confidence in the use of real-world assessments of progression and response as efficacy endpoints.

**Trial registration** NCT01740427; Funding: Pfizer.

## Introduction

Real-world evidence is playing an increasingly important role in regulatory decision-making, drug development, and clinical practice. [1–4] Because less than 5% of cancer patients participate in randomized clinical trials, [5] real-world evidence can provide valuable information on disease course and treatment outcomes of patients receiving care in front-line routine clinical settings, as well as insights on the generalizability of clinical trial findings to real-world patient populations. [3, 4, 6]

Real-world evidence is generated from real-world data documented during the course of routine clinical care. [2, 6–8] Real-world data can be derived from a range of sources, including electronic health records (EHRs), patient/disease registries, mobile devices and applications, genomic datasets, and medical/pharmacy claims databases. [2–4, 7, 8] Although these resources contain a wealth of information, they are designed to support clinical care and practice management, not clinical research. [4] Unlike randomized clinical trials (RCTs), which limit variability and ensure the quality of data collected through strict protocols and standardized methods such as case report forms, real-world datasets are typically disorganized and unstructured, requiring complex curation in order to be useful for research analyses. The quality and consistency of data in real-world sources, such as EHRs, can vary widely depending on the data curation processes used as well as on clinician-, practice-, and patient-related factors. These discrepancies can make it difficult to compare data collected in real-world settings with those from controlled clinical trials. [2, 8]

The most common outcome variables in cancer research are overall survival and assessments of tumor burden such as tumor response rate or progression free survival (PFS). [9, 10] In traditional RCTs, clinical response or disease progression is determined based on quantitative assessments of target lesions using a predefined scale (eg, Response Evaluation Criteria in Solid Tumors [RECIST]), applied at predefined time points (eg, every 6 to 8 wk), using prespecified imaging modalities (eg, computed tomography [CT] or magnetic resonance imaging [MRI]). [9] In clinical practice, assessments of tumor response and progression are based on clinician interpretations of imaging reports and symptomatic criteria. [9] (**Table 1**).

**Table 1:** Outcomes assessments and endpoints.

	Real-World Cohort	PALOMA-2 Cohort
**Tumor assessment interval**	Per clinical practice	Every 12 weeks
**Endpoints**	rwPFS^a^ rwRR^c^	PFS (RECIST v1.1)^b^ ORR (RECIST v1.1)^d^
**Data source(s)**	Clinician notes Radiology reports Pathology reports	Imaging (CT and/or MRI) Bone scans

CR, complete response; CT, computed tomography; MRI, magnetic resonance imaging; ORR, objective response rate; PFS, progression-free survival; PR, partial response; RECIST, Response Evaluation Criteria in Solid Tumors; rwCR = real-world complete response; rwPFS, real-world progression-free survival; rwPR = real-world partial response; rwRR, real-world response rate.

^a^Time from start of first-line letrozole monotherapy to clinically confirmed disease progression or death.

^b^Time from randomization to radiologically confirmed disease progression (per RECIST) or death.

^c^Maximum therapeutic response of rwCR or rwPR per treating clinician.

^d^Confirmed CR or PR per RECIST.

To evaluate the relationship between real-world and clinical trial outcomes in oncology, it is critical to assess the comparability of the data derived in each of these settings while minimizing the effect of confounding due to differences in prognostically important variables. [11, 12] The primary objective of this study was to compare PFS and response rates generated using real-world data reflecting routine clinical care with outcomes observed in a traditional RCT. To achieve this goal, we analyzed data from a curated EHR-derived real-world dataset to compare outcomes in a cohort of women with hormone-receptor positive (HR+), human epidermal growth factor receptor 2-negative (HER2–) metastatic breast cancer (mBC) who received first-line letrozole therapy in a real-world setting with those in the control arm of the phase 3 PALOMA-2 trial. [13] An inverse probability of treatment weighting (IPTW) approach was used to account for potential baseline differences in the real-world and PALOMA-2 cohorts, which allowed retrospective evaluation of the comparability of the real-world and traditional RECIST-based clinical trial endpoints in 2 similar cohorts. [14–16]

## Design and methods

### Study design and patients

The real-world cohort was drawn from de-identified patient data from the Flatiron Health database, a longitudinal, demographically and geographically diverse database derived from EHR data. [17] At the time of this study, the overall database encompassed more than 2000 clinicians at approximately 775 sites of care across the United States (US), representing 1.7 million patients with active cancer. Data for this study were derived from a curated subsample of patients with confirmed mBC.

The Flatiron real-world dataset is covered under the Health Insurance Portability and Accountability Act of 1996 (HIPAA) through Business Associate Agreements with every provider in the Flatiron network. These agreements authorize Flatiron to collect and de-identify patient-level structured and unstructured data to create de-identified data sets for research purposes. Processed data are de-identified according to either the Safe Harbor or Expert Determination method as outlined in HIPAA Section 164.514(b). When using the Expert Determination method, Flatiron employs a third-party expert to design the de-identification methodology and certify that the dataset is de-identified. Only de-identified data is delivered to clients. Institutional Review Board (IRB) approval was obtained for this study; informed consent was waived by the IRB as the study was retrospective and non-interventional, using routinely collected data. Details on the IRB are available in S1 Appendix.

**Fig 1:**
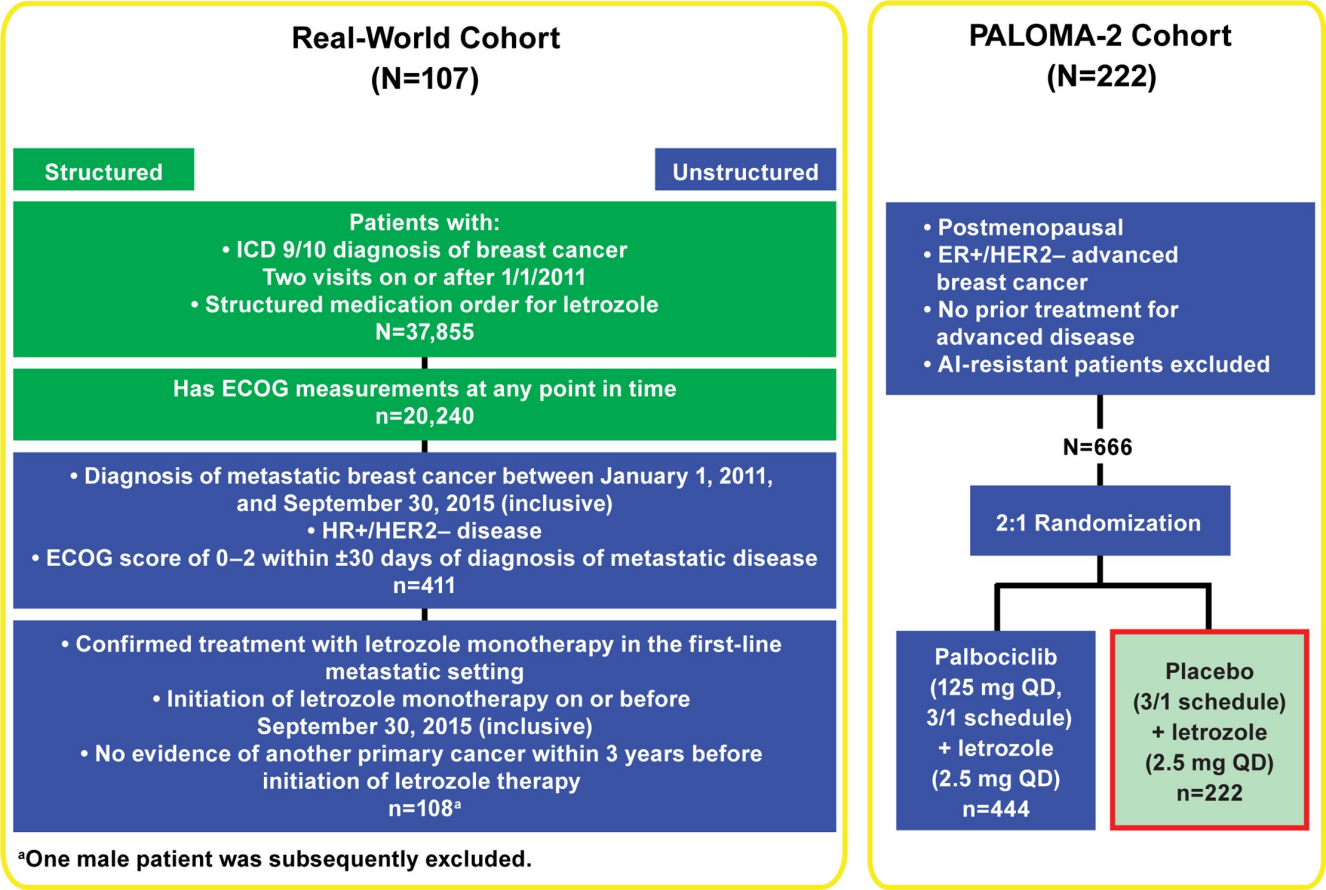
Design and consort diagram. ECOG PS = Eastern Cooperative Oncology Group performance status; ER = estrogen receptor; HER2- = human epidermal growth factor receptor 2-negative; ICD = International Classification of Diseases.

Data were derived from a random sampling (with attrition at each step, **Fig 1**) of women diagnosed with mBC between January 1, 2011, and September 30, 2015 (inclusive), regardless of menopausal status. Data provided were de-identified and provisions were in place to prevent re-identification in order to protect patients’ confidentiality. Eligibility criteria aligned with those of the PALOMA-2 trial and included documented HR+ (estrogen receptor–positive [ER+] or progesterone receptor–positive) and HER2– disease at any point before or ≤60 days following mBC diagnosis, an Eastern Cooperative Oncology Group performance status (ECOG PS) score <3 within 30 days of mBC diagnosis, and initiation of letrozole monotherapy in the first-line metastatic setting before October 1, 2015. Patients who had received previous treatment with a cyclin-dependent kinase 4/6 inhibitor or who had another primary cancer diagnosis ≤3 year before initiation of letrozole monotherapy were excluded.

The RCT cohort comprised women from the control arm of the double-blind, randomized, placebo-controlled, international, multicenter, phase 3 PALOMA-2 study (NCT01740427) (**Fig 1**). The study was approved by an IRB or equivalent ethics committee at each participating site, and all patients provided written informed consent before enrollment. Details on participating IRBs/ethics committees are available in S1 Appendix. The study was conducted in accordance with the International Conference on Harmonisation Good Clinical Practice guidelines and the provisions of the Declaration of Helsinki.

Eligible patients were postmenopausal women aged ≥18 years with ER+/ HER2– advanced breast cancer who had not received previous endocrine therapy for advanced disease. Inclusion criteria included postmenopausal status defined as previous bilateral surgical oophorectomy, spontaneous cessation of regular menses for 12 consecutive months, or follicle-stimulating hormone and estradiol blood levels in the respective postmenopausal ranges; adequate organ function; Eastern Cooperative Oncology Group performance status of 0−2; and measurable disease as defined per Response Evaluation Criteria in Solid Tumors (version 1.1). Exclusion criteria included HER2+ tumors; advanced, symptomatic, visceral spread at risk of life-threatening complications; previous neoadjuvant or adjuvant treatment with a nonsteroidal aromatase inhibitor with disease recurrence while on or within 12 months of completing treatment; and previous cyclin-dependent kinase 4/6 inhibitor treatment. [13]

### Treatment

In the real-world cohort, patients were treated with letrozole monotherapy per approved labeling and treating physicians’ clinical judgment. In the RCT cohort, women received letrozole (2.5 mg once daily, administered orally) plus placebo per the PALOMA-2 study protocol. [13]

### Endpoints and assessment

For the real-world cohort, tumor burden was assessed during routine clinical visits for patients with HR+/HER2− mBC. [9, 18] Tumor burden assessments were at the discretion of the treating physician and formalized RECIST methodology was not generally employed. Structured and unstructured patient-level data were extracted from the EHR using Flatiron Health’s proprietary technology-enabled abstraction platform, an electronic interface mimicking a case report form with centralized management and quality controls. This layer of technology facilitates document classification and visual organization, text search within documents, and selective presentation of relevant documents to trained data abstractors (clinical oncology nurses and tumor registrars). Structured data such as diagnoses, lab values, and medication administrations were mapped to a common terminology and unstructured data (eg, physician notes, lab/radiology reports) underwent manual review [19]. All abstractors received training in the use of the platform as well as indication-specific training (operating procedures, best practice guidelines) prior to beginning the abstraction process.

Curated progression events were designated “real-world progression” (rwP). The approach to rwP anchors on clinician-documented cancer progression based on an interpretation of the entire patient chart, including results of diagnostic procedures and tests (eg, radiology and pathology reports). [19] The date of cancer progression was defined as the date of the first source evidence for progression referenced by the clinician (eg, radiology report date) or the date of clinician note when no other corresponding evidence sources were documented. A parallel construct reflecting real-world progression-free survival (rwPFS) was calculated, measuring from the start of first-line letrozole therapy through the end of first-line therapy for patients receiving only first-line therapy and to the start of second-line therapy for all other patients. Patients without disease progression or death were censored at the end of first-line therapy (patients who only received first-line therapy) or at the start of second-line therapy (all others). The approach to real-world response is based on clinician-documented assessments of radiologic change in burden of disease over the course of treatment with a given therapy. Real-world response rate (rwRR) was calculated as the percentage of patients in the cohort with a maximum clinician-assessed therapeutic response of complete response (rwCR) or partial response (rwPR) (**Table 2**).

**Table 2:** Real-world response categories vs RECIST v1.1 [9].

Response Category	Real-world^a^	RECIST v1.1^b^
**Complete response**	Complete resolution of all visible disease	Disappearance of all target lesions; reduction in short axis of pathologic lymph modes to <10 mm
**Partial response**	Partial reduction in size of visible disease in some or all areas without any areas of increase in visible disease	≥30% decrease from baseline in the sum of the diameters of all target measurable lesions
**Stable disease**	No change in overall size of visible disease (includes cases where some lesions increased and some lesions decreased in size)	Insufficient change to qualify for PR, CR, or PD
**Progressive disease**	Increase in visible disease or presence of new lesions	Appearance of ≥1 new lesion or ≥20% increase from the smallest sum of target lesions documented on study with a minimum increase of 5 mm
**Indeterminate/equivocal**	Clinician specifically indicates that response is “indeterminate” or “uncertain” or if clinician’s interpretation of the scan(s) cannot be mapped to 1 of the above categories	Necrosis or cystic changes in target lesions, very small or uncertain new lesions

Abbreviations: CR, complete response; PD, progressive disease; PR, partial response; RECIST, Response Evaluation Criteria in Solid Tumors; SD, stable disease.

^a^Assessment of visible disease.

^b^Assessment of all target lesions (lesions measurable at baseline up to a maximum of 5 lesions total and 2 lesions per organ).

For the PALOMA-2 cohort, tumor assessment (CT with contrast or MRI) was conducted every 12 weeks +/−7 days for patients with measurable disease; patients with bone-only disease received bone scans every 6 months. [13] Imaging and bone scans were performed until objective disease progression, initiation of a new anticancer therapy, or withdrawal from the study, whichever came first. [13] PFS and ORR were measured per RECIST version 1.1 (**Table 2**). PFS was defined as the time from the date of randomization to the date of radiologically confirmed disease progression or death due to any cause, whichever occurred first, and calculated using a similar approach as described for rwPFS; ORR was estimated by dividing the number of patients with confirmed CR or PR by the number of patients randomized to letrozole plus placebo with measurable disease at baseline. [13]

Of note, in PALOMA-2 all deaths that occurred through 28 days after the end of first-line therapy were included as progression events. In the real-world cohort, however, death dates were reported only by month and year. To align progression definitions, patients in the real-world cohort who died in the same month or within one month of the stop date of first-line therapy were included as progression events.

### Analysis and statistical methods

Inverse probability of treatment weighting was used to adjust analyses for differences in observed potential confounders between the 2 study cohorts. [16, 20, 21] The IPTW process modifies the patient counts according to differences in unweighted baseline characteristics.

Propensity scores were generated using a multivariable logistic model executed on data from 107 real-world patients and 222 PALOMA-2 patients. Study origin (real-world or PALOMA-2) was used as an outcome and potential baseline confounders were included as covariates, having been selected based on the authors’ clinical judgment. Covariates included were age, race, disease stage at diagnosis (I–IV or unrecorded/unknown), ECOG PS score, number of disease sites at diagnosis (1, 2, ≥3), and bone-only metastases. In order to balance the 2 study cohorts for duration of follow-up, the propensity score model also included potential follow-up, a baseline measure defined as the number of months from a patient’s start of treatment date to the study cutoff: September 30, 2016 for the real-world cohort and February 26, 2016 for PALOMA-2.

Inverse probability of treatment weights were then generated for each patient by inverting their propensity score and stabilizing the score to reduce influences from large weights (small propensity scores) by multiplying the inverted propensity score by 107/329 for Flatiron patients and 222/329 for PALOMA-2 patients. The balance in prognostically important baseline characteristics was assessed using a standardized differences approach, with values ≥0.10 indicating a non-negligible imbalance.

The duration of first line letrozole therapy was abstracted using Flatiron business rules applied to patient EHRs for the real-world cohort. For the PALOMA-2 cohort, the duration of treatment was obtained from information recorded in the data collection tool used in the study.

The Kaplan-Meier method was used to estimate median rwPFS and RECIST-based PFS for the real-world and PALOMA-2 cohorts, respectively. Hazard ratios and 95% confidence interval (CI) were computed using weighted Cox proportional hazards analysis with IPTW adjustment. A 2-sided *p* < 0.05 was considered significant. All statistical analyses were performed using SAS v.9.4.

## Results

### Patient population

Between January 1, 2011, and September 30, 2015 (data cutoff, September 30, 2016), 107 women initiated letrozole monotherapy and met the eligibility criteria for inclusion in the unadjusted real-world cohort (**Fig 1**). In PALOMA-2, 222 patients were randomized to treatment with letrozole plus placebo between February 2013 and July 2014 (cutoff date for final analysis, February 26, 2016) and were included in the unadjusted RCT cohort (**Table 3**). The number of patients in each cohort were modified by IPTW according to differences in unweighted baseline characteristics. Rounding to the nearest whole number, the IPTW-adjusted number was 116 for the real-world cohort and 207 for the RCT cohort (**Table 4**).

**Table 3:** Baseline demographic and clinical characteristics (Unweighted).

Characteristic	Real-World Cohort (N = 107)	PALOMA-2 Cohort (N = 222)
**Female, n (%)**	107 (100)	222 (100)
**Age, y**		
Mean (SD)	68.6 (11.1)	60.6 (11.2)
Median (range)	69 (34−84)	61 (28−88)
<65	36 (33.6)	141 (63.5)
≥65	71 (66.4)	81 (36.5)
**Race, n (%)**		
White	67 (62.6)	172 (77.5)
Black or African American	8 (7.5)	3 (1.4)
Asian	2 (1.9)	30 (13.5)
Other	30 (28.0)	17 (7.7)
**ECOG PS, n (%)**^**a**^		
0	61 (57.0)	102 (46.0)
1	33 (30.8)	117 (52.7)
2	13 (12.1)	3 (1.4)
**Stage at diagnosis, n (%)**^**b**^		
I	22 (20.6)	30 (13.5)
II	21 (19.6)	68 (30.6)
III	10 (9.3)	39 (17.6)
IV	42 (39.3)	72 (32.4)
Stage not recorded/unknown	12 (11.2)	13 (5.9)
**Number of involved disease sites**		
1	41 (38.3)	66 (29.7)
2	40 (37.4)	52 (23.4)
≥3	26 (24.3)	104 (46.8)
Bone-only metastases, n (%)	32 (29.9)	48 (21.6))
**Menopausal status, n (%)**		
Premenopausal	5 (4.7)	0
Postmenopausal	67 (62.6)	222 (100)
Unknown	35 (32.7)	0
Age <60 y, n (%)	1 (2.9)	NA
Age ≥60 y, n (%)	34 (97.1)	NA
**Hormone receptor status, n (%)**^**d**^		
ER+	107 (100)	222 (100)
PR+	76 (71.0)	NA
PR−	19 (17.8)	NA
Unknown	12 (11.2)	NA
**HER2 status, n (%)**		
Negative	105 (98.1)	222 (100)
Equivocal^e^	2 (1.9)	0

ECOG PS, Eastern Cooperative Oncology Group performance status; ER, estrogen receptor; HER2, human epidermal growth factor receptor 2; IPTW, inverse probability of treatment weighting; NA, data not available; PR, progesterone receptor; RCT, randomized controlled trial; RW, real-world; SD, standard deviation.

^a^Includes not reported/missing patients

^b^For the real-world cohort, the lowest ECOG PS score for each patient within the 30-day index window is reported.

^c^Disease stages do not include IB, IIA and IIB, or IIIB and IIIC classifications.

^d^For the real-world cohort, the result depicts the test closest to the metastatic diagnosis date, that is, within ±60 days of the metastatic diagnosis date.

^e^Despite confirmation of HER2− status before metastatic diagnosis, a test closer to the metastatic diagnosis date was equivocal, and the most recent result was documented.

**Table 4:** Demographic and clinical characteristics of interest, before and after IPTW adjustment.

Characteristic	Before IPTW			After IPTW		
	Real-World Cohort (N = 107)	PALOMA-2 Cohort (N = 222)	Standardized Difference^a^	Real-World Cohort (N = 116)^b^	PALOMA-2 Cohort (N = 207)^b^	Standardized Difference^a^
**Female, %**	100	100	0.0000	100	100	0.0000
**Age, y, mean (SD)**	68.6 (11.1)	60.6 (11.2)	0.7175	62.3 (12.7)	62.5 (11.0)	0.0222
**Race, n (%)**						
White	67 (62.6)	172 (77.5)	0.3288	74.8 (64.6)	162.9 (78.5)	0.3132
Black or African American	8 (7.5)	3 (1.4)	0.3016	5.3 (4.5)	2.8 (1.4)	0.1892
Asian	2 (1.9)	30 (13.5)	0.4478	2.1 (1.8)	26.6 (12.8)	0.4333
Other	30 (28.0)	17 (7.7)	0.5521	33.7 (29.1)	15.2 (7.3)	0.5888
**ECOG PS**^**c**^**, n (%)**						
0	61 (57.0)	102 (46.0)	0.2227	62.1 (53.6)	104.9 (50.6)	0.0610
1	33 (30.8)	117 (52.7)	0.4546	48.1 (41.5)	93.6 (45.1)	0.0729
2	13 (12.1)	3 (1.4)	0.4407	5.6 (4.9)	8.9 (4.3)	0.0270
**Stage at diagnosis**^**d**^**, n (%)**						
I	22 (20.6)	30 (13.5)	0.1883	21.4 (18.5)	30.5 (14.7)	0.1023
II	21 (19.6)	68 (30.6)	0.2558	25.9 (22.4)	61.1 (29.5)	0.1623
III	10 (9.3)	39 (17.6)	0.2427	15.9 (13.7)	30.5 (14.7)	0.0274
IV	42 (39.3)	72 (32.4)	0.1426	43.1 (37.2)	72.6 (35.0)	0.0470
Not recorded/unknown	12 (11.2)	13 (5.9)	0.1927	9.5 (8.2)	12.9 (6.2)	0.0769
**Involved disease sites, n (%)**						
1	41 (38.3)	66 (29.7)	0.1820	39.8 (34.4)	63.0 (30.4)	0.0854
2	40 (37.4)	52 (23.4)	0.3070	34.8 (30.1)	62.9 (30.3)	0.0064
≥3	26 (24.3)	104 (46.8)	0.4846	41.2 (35.6)	81.5 (39.3)	0.0765
Bone-only metastases	32 (29.9)	48 (21.6)	0.1903	26.9 (23.2)	44.6 (21.5)	0.0422
**Potential follow-up, mo**^**e**^ **(SD)**	33.0 (13.6)	24.5 (3.3)	0.8544	25.2 (11.2)	25.1 (3.6)	0.0128

Abbreviations: ECOG PS, Eastern Cooperative Oncology Group performance status; ER, estrogen receptor; HER2, human epidermal growth factor receptor 2; IPTW, inverse probability of treatment weighting; PR, progesterone receptor; SD, standard deviation.

^a^The threshold for ignorable differences is 0.10.

^b^IPTW-adjusted according to differences in unweighted baseline characteristics.

^c^For the real-world cohort, the lowest ECOG PS score for each patient within the 30-day index window is reported.

^d^Disease stages do not include IB, IIA and IIB, or IIIB and IIIC classifications.

^e^From start of treatment to study cutoff (September 30, 2016 for real-world cohort; February 26, 2016 for PALOMA-2)

Unweighted, unadjusted demographic and clinical characteristics of the 2 cohorts were broadly comparable, although patients in the real-world cohort were older (mean age 68.6 vs 60.6 y in PALOMA-2), more racially diverse, had poorer performance status (12.1% vs 1.4% with ECOG PS 2), and were more likely to have stage IV disease (39.3% vs 32.4%) and bone-only metastases (29.9% vs 21.6%) at diagnosis (**Table 3**). Of note, two patients with confirmed HER2− disease prior to their metastatic diagnosis had equivocal results when tested closer to the metastatic diagnosis date. In both cases the most recent result was documented.

Data abstractors were instructed to record menopausal status only when it was explicitly stated in the patient’s chart. As a result, more than one-third of patients were classified as “unknown.” As all but 1 of these patients—a 54 year old—were over the age of 60, these patients were retained in the real-world dataset (**Table 3**). Five patients classified as “premenopausal” were also retained. Because letrozole is specifically contraindicated in women of premenopausal status it could reasonably be inferred that these patients met the criteria for medically confirmed postmenopausal status or were in medically-induced menopause as a result of ovarian suppression per current treatment guidelines and standard practice.

After IPTW, standardized differences were reduced for all baseline demographic and clinical variables of interest. Standardized differences were <0.10 for prognostically important variables including age, ECOG PS, disease stage III or IV, bone-only metastases, and potential follow-up (**Table 4**). Standardized differences for disease stage I and II were <0.20 (**Table 4**).

### PFS and treatment duration

**Fig 2:**
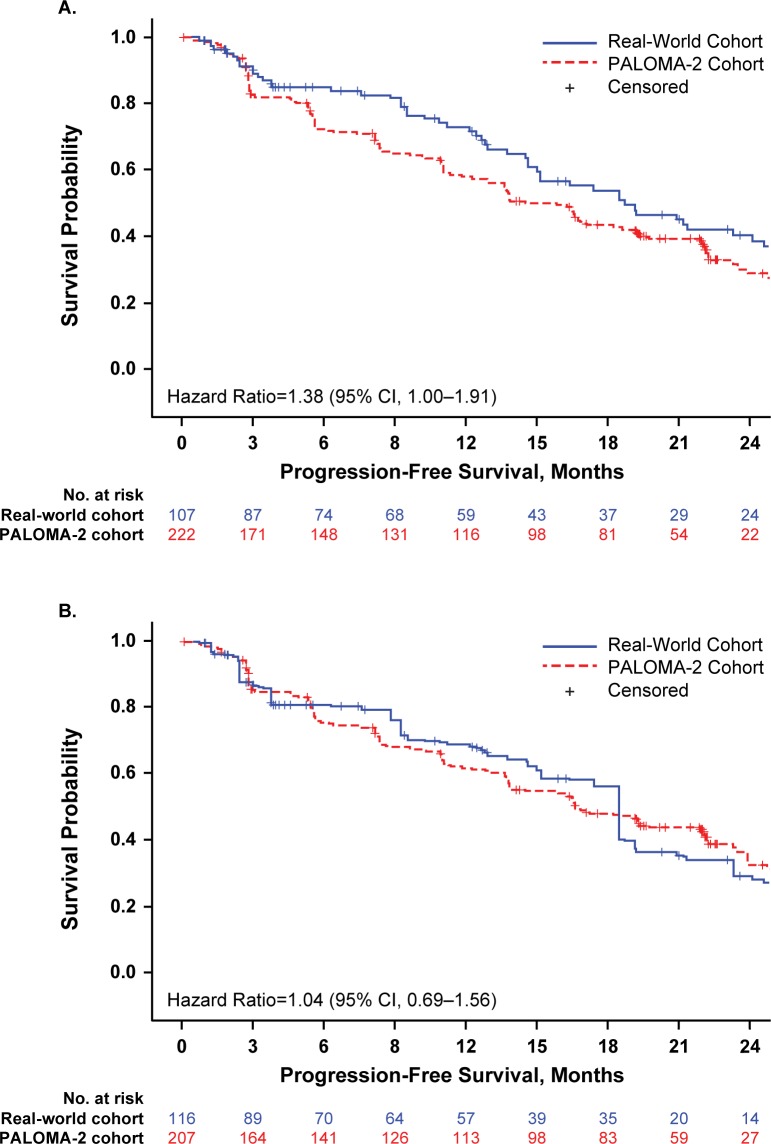
Unadjusted (A) and IPTW-Adjusted (B) Progression-Free Survival. CI, confidence interval; IPTW, inverse probability of treatment weighting. IPTW adjusted numbers of patients at risk are shown.

Using unadjusted and unweighted patient data for the 2 cohorts, median rwPFS was 18.7 months (95% CI, 14.6–24.1) for real-world patients and PFS was 14.5 months (95% CI, 12.9–17.1) for PALOMA-2 patients (hazard ratio, 1.38 [95% CI, 1.00‒1.91]); **Fig 2A**). Median rwPFS was longer than PFS in the PALOMA-2 cohort, potentially reflecting the higher proportion of patients with bone-only disease in this group. Following IPTW adjustment, median PFS was similar in both cohorts: 18.4 months (95% CI, 12.8–23.3) for the real-world group and 16.6 months (95% CI, 13.7–22.2) for the PALOMA-2 group (**Fig 2B**). The hazard ratio using real-world data as reference was 1.04 (95% CI, 0.69–1.56).

The unweighted, unadjusted mean (standard deviation [SD]) duration of first-line letrozole treatment was slightly longer among patients in the real-world cohort than in the PALOMA-2 cohort: 17.1 months (13.0) and 14.0 months (8.9), respectively, standardized difference, 0.2810. After IPTW-adjustment, mean (SD) duration of treatment was 13.3 months (11.1) in the real-world cohort and 14.6 months (8.9) in the PALOMA-2 group, with a reduction in standardized difference to 0.1242 (**Table 5**). Discontinuations due to treatment-related adverse events or toxicity were relatively low, and were reported more frequently among patients in the real-world cohort than in the PALOMA-2 cohort (6.5% and 4.1%, respectively) (**Table 6**).

**Table 5:** Duration of first-line letrozole therapy, before and after IPTW adjustment.

	Before IPTW			After IPTW		
	Real-World Cohort^a^ (N = 107)	PALOMA-2 Cohort^b^ (N = 222)	Standardized Difference^c^	Real-World Cohort^a^ (N = 116)^d^	PALOMA-2 Cohort^b^ (N = 207)^d^	Standardized Difference^c^
**Duration, mean, mo (SD)**	17.1 (13.0)	14.0 (8.9)	0.2810	13.3 (11.1)	14.6 (8.9)	0.1242

IPTW, inverse probability of treatment weighting; SD = standard deviation.

^a^ Calculated as days from start of treatment up to the last clinic note/death date.

^b^ Defined as the total number of dosing days from first to last day (inclusive) of each study treatment, divided by 30.44 to convert to months.

^c^The threshold for ignorable differences is 0.10.

^d^IPTW-adjusted according to differences in unweighted baseline characteristics.

**Table 6:** Reasons for discontinuation (Unweighted).

Reasons for Discontinuation	Real-World Cohort (N = 107)	PALOMA-2 (N = 222)
**Total discontinued, n (%)**	49 (45.8)	161 (72.5)
Disease progression or death	36 (33.6)	127 (57.2)
Treatment-related AE/toxicity	7 (6.5)	9 (4.1)
Other^a^	4 (3.7)	22 (9.9)
Protocol violation	NA	3 (1.4)
Unknown	2 (1.9)	NA

AE = adverse event; NA, not applicable; SD = standard deviation.

^a^For patients in the real-world cohort, the reasons were as follows: other (n = 3 [2.8%]), patient request unrelated to toxicity or financial issues (n = 1 ([0.9%]). For patients in the PALOMA-2 cohort, the reasons were as follows: global deterioration of health status(n = 9 [4.1%]), refused to continue for reason other than AE (n = 9 [4.2%]), other (n = 4 [1.8%]).

### Tumor response

**Fig 3:**
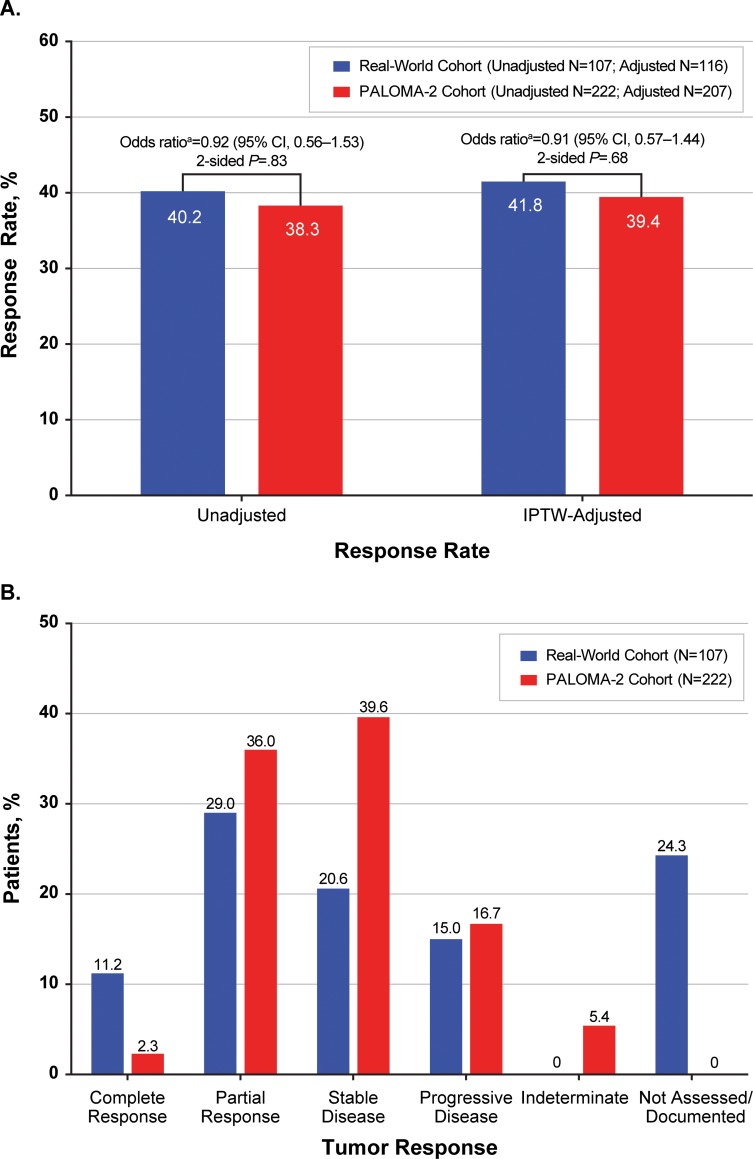
Summary of Response Rate (A) and Tumor Response (B) in Patients Receiving First-Line Letrozole for HR+/HER2– mBC. CI, confidence interval; HER2–, human epidermal growth factor receptor 2-negative; HR+, hormone-receptor positive; IPTW, inverse probability of treatment weighting; mBC, metastatic breast cancer; RWD, real-world data. ^**a**^RWD as reference.

Using unadjusted and unweighted patient data, the rwRR in the real-world cohort (40.2% [95% CI, 30.8–50.1]) was similar to the ORR in the PALOMA-2 cohort (38.3% [31.9–45.0]; odds ratio: 0.92 [95% CI, 0.56–1.53]; 2-sided *P* = .83). No significant difference was observed between rwRR and ORR in IPTW adjusted comparisons: 41.8% and 39.4%, respectively (odds ratio: 0.91 [95% CI, 0.57–1.44]; 2-sided *P* = .68; **Fig 3A**). Complete tumor response was more frequently reported in the unadjusted real-world cohort (11.2%) than the unadjusted PALOMA-2 group (2.3%) (**Fig 3B**). Of note, 22.4% of patients in the real-world cohort had no tumor assessments recorded during a mean 5.8 months of first line therapy.

## Discussion

To our knowledge, this is the first study in oncology clinical research to establish concordance on time-dependent efficacy endpoints between real-world and RCT datasets. Our analysis found that after IPTW adjustment for potentially confounding demographic and clinical characteristics, tumor burden endpoints such as rwPFS and rwRR derived from curated real-world data were similar to those observed in an RCT in women treated with letrozole monotherapy as first-line treatment for HR+/HER2– mBC. Median rwPFS in the real-world cohort was 18.4 months versus a median PFS of 16.6 months in the PALOMA-2 cohort, with a rwRR of 41.8% versus an ORR of 39.4% in PALOMA-2 patients.

As the number of novel oncology therapies entering the market increases, the need to assess the efficacy of these therapies relative to one another will become increasingly important. Reliable real-world data can help efficiently address this growing demand. There is a growing interest in the use of real-world data to support modern clinical trial design. Real-world data can facilitate the study of new agents in populations that are more reflective of the diverse patients encountered in routine clinical practice, either as internal control arms or as external control arms for single-arm trials. [1–4] At the regulatory level, single-arm trials with surrogate endpoints supported by external control data could be the basis for rapid approval of novel agents with exceptional clinical activity, while high quality phase IV studies in the real-world setting could provide confirmatory evidence following accelerated approvals. [2]

If real-world data are to be integrated into clinical trials, increasing confidence in the validity of real-world endpoints is critical. Conventional RECIST-based assessment relies on quantitative measurement of target lesions with consistent imaging modality and strict assessment intervals. In real-world clinical practice, the assessment of progression or treatment response is often qualitative and based on diverse clinical factors, including imaging studies, clinical presentation, and patient-specific factors such as performance status.

This analysis demonstrated consistency between rwPFS/rwRR and RECIST-based correlates despite these fundamental differences. A key advantage of this work was that the endpoints were subjected to similar analytic conditions as would be expected for traditional clinical trial endpoints and performed similarly. Available individual patient-level data from the PALOMA-2 cohort allowed for patient-level weighting of study populations and increased confidence in results.

There were several limitations of this analysis. Differences in clinical and sociodemographic characteristics were observed between the real-world and PALOMA-2 cohorts that confirm the well-established observation that patients who enroll in RCTs tend to be younger, healthier, and less racially and ethnically diverse than the general population of cancer patients. [5] Inverse probability of treatment weighting was used to control for these imbalances, and IPTW-adjusted baseline characteristics were comparable between the 2 cohorts. However, IPTW cannot completely overcome initial selection bias and does not control for unobserved confounders; as a result, unmeasured confounding may still be present even in the weighted observations.

In addition, although the inclusion/exclusion criteria for the real-world cohort were designed to align with those of PALOMA-2 as much as possible, there were differences in selection criteria between the 2 groups. In PALOMA-2, prior adjuvant or neoadjuvant therapy with a nonsteroidal AI was allowed unless disease recurred while the patient was on therapy or within 12 months of therapy completion. While start and stop dates of other endocrine therapies were abstracted from unstructured chart data in the real-world cohort, it was not feasible to determine timing relative to disease recurrence, so patients with a history of prior AI therapy were not excluded. However, it could reasonably be inferred that treating physicians followed current treatment guidelines, which recommend that patients who received prior endocrine therapy within 1 year of recurrence be treated with a different endocrine therapy.

Similarly, postmenopausal status was an eligibility criterion in the prospective PALOMA-2 trial, but was not a requirement for inclusion in the retrospective real-world cohort. In the PALOMA-2 study, rigorous screening criteria were in place to ensure all enrolled patients were postmenopausal. [13]. In routine clinical practice, however, menopausal status often goes undocumented in the EHR. In the real-world dataset, menopausal status was recorded only when explicitly stated in the patient’s chart and age was not used as a proxy. As a result, approximately one-third of patients in the real-world cohort had a menopausal status of “unknown.” All of these patients were over the age of 50, and all but 1 was over the age of 60. The differences in menopausal status were partially adjusted for by the inclusion of age as a variable in the computation of the weights in the IPTW process—as indicated by a change in standardized difference from 0.7175 before IPTW adjustment to 0.0222 after (**Table 4**).

The real-world patients in this analysis had longer unadjusted rwPFS, possibly due to the higher proportion of bone-only metastases—which is a potential indicator of more indolent disease. [22, 23]—in the real-world cohort. The between-groups difference was substantially reduced following IPTW adjustment (HR = 1.04; 95% CI 0.69–1.56).

In addition, in contrast to the global PALOMA-2 study, patients in the real-world cohort are all from the US and receive care in routine clinical settings, which may have contributed to the observed differences in the frequency of tumor assessments. The PALOMA-2 protocol specified that tumor assessments be conducted every 12 weeks, while in the real-world cohort scans were ordered at the discretion of the treating physician. It is noteworthy that 12 of the patients without tumor assessments had durations of treatment longer than 12 weeks.

Despite these limitations, this analysis increases confidence that data from real-world health care databases can be used to match the populations of randomized clinical trials and to assess key outcomes in clinical practice settings. Although this is, to our knowledge, the first study of its kind in oncology, a similar analysis of data from a large health care database successfully mirrored the composite endpoints of the pivotal ONTARGET trial of the angiotensin receptor blocker telmisartan. [24] The analysis of data from more than 50,000 patients took approximately 12 weeks at a fraction of the cost of the pivotal trial. [24]

Deriving endpoints in the oncology setting is admittedly a more labor-intensive task, requiring manual review of unstructured chart elements (eg, clinician notes, radiology reports) to arrive at high quality clinical outcome data. Enhancing the interoperability of EHRs and improving the capture of outcomes data are core goals of regulatory and private sector efforts to promote meaningful use of health information technology (HIT). [25] Modifying EHRs to include structured fields that capture progression and response and training clinicians to enter relevant data into the correct fields may provide an easier path to capturing outcome measures in oncology clinical practice settings, and facilitate both retrospective and prospective studies of real-world outcomes. Such an effort would require the coordinated efforts of multiple stakeholders to provide the necessary HIT framework, education, and support to physicians and allied health professionals.

## Conclusions

This study is a preliminary but important step in showing that clinically meaningful information can be derived from the assessment of rwPFS and rwRR based on EHR data abstraction when proper quality controls and analytic methods are incorporated. Although limited to patients with mBC, the current study lays the groundwork for additional analyses that could be used to investigate treatment effects using real-world data in other malignancies. With further validation, real-world data may help to modernize the clinical trial landscape and enhance the design of prospective real-world randomized studies.

## Supporting information

S1 Appendix List of independent ethics committees or institutional review boards. (DOCX)
