# Supplementary material for: Concordance of real-world versus conventional progression-free survival from a phase 3 trial of endocrine therapy as first-line treatment for metastatic breast cancer
Source: PLoS One. 2020 Apr 21;15(4):e0227256. doi: 10.1371/journal.pone.0227256 (PMC7173855; doi:10.1371/journal.pone.0227256)
Supplement: S1 Appendix — (DOCX) [file pone.0227256.s001.docx]

# S1 Appendix - List of Independent Ethics Committees or Institutional Review Boards

# Flatiron:

Copernicus Group Independent Review Board (CGIRB)

5000 CentreGreen Way, Suite 200

Cary NC 27513

# PALOMA-2:

| **Study Site Number** | **Independent Ethics Committee or Institutional Review Board Address(es)** | |
| --- | --- | --- |
| **AUSTRALIA** | | |
| 1150 | Hunter New England Human Research Ethics Committee  Locked Bag No. 1  New Lambton, NSW 2305  AUSTRALIA | |
| 1151 | Hunter New England Human Research Ethics Committee  Locked Bag No. 1  New Lambton, NSW 2305  AUSTRALIA | |
| 1152 | Hunter New England Human Research Ethics Committee  Locked Bag No. 1  New Lambton, NSW 2305  AUSTRALIA | |
| 1153 | Hunter New England Human Research Ethics Committee  Locked Bag No. 1  New Lambton, NSW 2305  AUSTRALIA | |
| 1154 | Epworth HealthCare Research and Ethics Committee  89 Bridge Road, Epworth Healthcare  Richmond, VIC 3121  AUSTRALIA | |
| 1155 | Hunter New England Human Research Ethics Committee  Locked Bag No. 1  New Lambton, NSW 2305  AUSTRALIA | |
| 1156 | Hunter New England Human Research Ethics Committee  Locked Bag No. 1  New Lambton, NSW 2305  AUSTRALIA | |
| 1157 | Bellberry Human Research Ethics Committee  129 Glen Osmond Road  Eastwood, SOUTH AUSTRALIA 5063  AUSTRALIA | |
| 1159 | Hunter New England Human Research Ethics Committee  Locked Bag No. 1  New Lambton, NSW 2305  AUSTRALIA | |
| 1160 | Hunter New England Human Research Ethics Committee  Locked Bag No. 1  New Lambton, NSW 2305  AUSTRALIA | |
| 1164 | Hunter New England Human Research Ethics Committee  Hunter New England Research Ethics and Governance Unit  Locked Bag 1  New Lambton, NSW 2305  AUSTRALIA | |
| 1166 | Hunter New England Human Research Ethics Committee  Locked Bag No. 1  New Lambton, NSW 2305  AUSTRALIA | |
| 1200 | Research Ethics Committee, Royal Adelaide Hospital  Level 3, Hansen Institute, IMVS Building, Royal Adelaide Hospital, North Terrace  Adelaide, SA 5000  AUSTRALIA | |
| 1279 | Royal Perth Hospital Human Research Ethics Committee  Royal Perth Hospital, Ethics Office, Level 5 Colonial House  Perth, WA 6001  AUSTRALIA | |
| **BELGIUM** | | |
| 1036 | Commissie Medische Ethiek van Universitaire Ziekenhuizen KU Leuven  Campus Gasthuisberg, Herestraat 49  Leuven, 3000  BELGIUM | |
| 1039 | Commissie Medische Ethiek van de Universitaire Ziekenhuis K.U. Leuven  Campus Gasthuisberg E330, Herestraat 49  Leuven, 3000  BELGIUM | |
| 1054 | COMMISSIE MEDISCHE ETHIEK VAN DE UNIVERSITAIRE ZIEKENHUIZEN KU LEUVEN  Campus Gasthuisberg, Herestraat 49  Leuven, B-3000  BELGIUM | |
|  | Comite Ethique CHU Sart Tilman  1, avenue de l'hopital/Domaine universitaire B35, Tour 2, niveau -2E  Liege, 4000  BELGIUM | |
| 1076 | Commissie Medische Ethiek van Universitaire Ziekenhuizen KU Leuven  Campus Gasthuisberg, Herestraat 49  Leuven  BELGIUM | |
| 1113 | COMMISSIE MEDISCHE ETHIEK VAN DE UNIVERSITAIRE ZIEKENHUIZEN KU LEUVEN  Campus Gasthuisberg, Herestraat 49  Leuven, B-3000  BELGIUM | |
| 1142 | Commissie Medische Ethiek van Universitaire Ziekenhuizen KU Leuven,  Campus Gasthuisberg, Herestraat 49,  Leuven, 3000  BELGIUM | |
| 1192 | Commissie Medische Ethiek van Universitaire Ziekenuizen KU Leuven  Campus Gasthuisberg, Herestraat 49  Leuven, 3000  BELGIUM | |
| 1262 | Commissie Medische Ethiek van Universitaire Ziekenuizen KU Leuven  Campus Gasthuisberg, Herestraat 49  Leuven, 3000  BELGIUM | |
| **CANADA** | | |
| 1074 | Comite d'ethique de la recherche et de l'Evaluation des Technologies de la Sante  5400 Gouin Boulevard West, Hopital du Sacre-Coeur  Montreal, QC H4J 1C5  CANADA | |
| 1205 | Centre for Applied Ethics, McGill University Health Centre (MUHC)  2155 Guy Street, Room 223.09  Montreal, QC H3H 2R9  CANADA | |
| 1219 | Comite d'ethique de la recherche du CHU de Quebec  10, rue de l'Espinay, Universite Laval  Quebec, QC G1L 3L5  CANADA | |
| 1220 | Ontario Cancer Research Ethics Board  661 University AvenueOntario Cancer Research Ethics Board, MaRS Centre Suite 510  Toronto, ON M5G 0A3  CANADA | |
| 1225 | Nova Scotia Health Authority Research Ethics Board  5790 University Avenue, Room 118  Halifax, NS B3H 1V7  CANADA | |
| 1226 | Health Research Ethics Board of Alberta - Cancer Committee  10104-103 Avenue NW, 1500  Edmonton, AB T5J4A7  CANADA | |
| 1231 | UBC BCCA Research Ethics Board (BCCA REB)  Fairmont Medical BuildingBC Cancer Agency, 750 West Broadway, Suite 902  Vancouver, BC V5Z 1H5  CANADA | |
| 1232 | Ontario Cancer Research Ethics Board  MaRS Centre  c/o Ontario Institute for Cancer Research, 661 University Avenue, Suite 510  Toronto, ON M5G 0A3  CANADA | |
| 1233 | Ontario Cancer Research Ethics Board  MaRS Centre, South Tower  C/o Ontario Institute for Cancer Research, 101 College Street, Suite 800  Toronto, ON M5G 0A3  CANADA | |
| 1239 | Ontario Cancer Research Ethics Board (OCREB)  661 University Avenue, MaRS Centre - Suite 510  Toronto, ON M5G 0A3  CANADA | |
| 1240 | Ontario Cancer Research Ethics Board  MaRS Centre, South Tower  C/o Ontario Institute for Cancer Research, 101 College Street, Suite 800  Toronto, ON M5G 0A3  CANADA | |
| 1241 | University of Saskatchewan BioMedical Research Ethics Board (Bio-REB)  1607 110 Gymnasium PlaceResearch Ethics Office, University of Saskatchewan, NRC, Plant Biotechnology Research Institute  Saskatoon, SK S7N 4J8  CANADA | |
| 1273 | Ontario Cancer Research Ethics Board  MaRS Centre, South Tower  101 College Street, Suite 800  Toronto, ON M5G 0A3  CANADA | |
| 1282 | UBC BCCA Research Ethics Board (BCCA REB)  Fairmont Medical BuildingBC Cancer Agency, 750 West Broadway, Suite 902  Vancouver, BC V5Z 1H5  CANADA | |
| 1291 | Health Research Ethics Board of Alberta - Cancer Committee  10104-103 Ave NWC/O Alberta Innovates - Health Solutions, 1500  Edmonton, AB T5J 4A7  CANADA | |
| **CZECH REPUBLIC** | | |
| 1097 | Eticka komise FN Olomouc  I.P.Pavlova 6  Olomouc, 775 20  CZECH REPUBLIC | |
|  | Eticka komise pro multicentricke klinicke hodnoceni FN Motol  Fakultni nemocnice Motol, V uvalu 84  Praha 5, 15006  CZECH REPUBLIC | |
| 1288 | Eticka komise pro multicentricke klinicke hodnoceni FN Motol  Fakultni nemocnice Motol, V uvalu 84  Praha 5, 15006  CZECH REPUBLIC | |
|  | Eticka komise  Sokolska 581, FN Hradec Kralove  Hradec Kralove, 500 05  CZECH REPUBLIC | |
| **FRANCE** |  | |
| 1037 | CPP Ile de France 3  89 rue d'Assas, Hopital Tarnier  Paris, 75006  FRANCE | |
| 1062 | CPP Ile de France 3  Hôpital Tarnier, 89, rue d’Assas  PARIS, 75006  FRANCE | |
| 1063 | CPP Ile de France 3  Hôpital Tarnier, 89, rue d’Assas  PARIS, 75006  FRANCE | |
| 1064 | CPP Ile de France 3  Hôpital TARNIER, 89, rue d’Assas  PARIS, 75006  FRANCE | |
| 1065 | CPP Ile de France 3  Hôpital TARNIER, 89, rue d’Assas  PARIS, 75006  FRANCE | |
| 1066 | CPP Ile de France 3  89 rue d'Assas, Hopital Tarnier  Paris, 75006  FRANCE | |
| 1182 | CPP Ile de France 3  Hôpital Tarnier Cochin, 89, rue d’Assas  PARIS, 75006  FRANCE | |
| 1183 | CPP Ile de France 3  Hôpital TARNIER, 89, rue d’Assas  PARIS, 75006  FRANCE | |
| 1285 | CPP Ile de France 3  Hôpital Tarnier Cochin, 89, rue d’Assas  PARIS, 75006  FRANCE | |
| 1293 | CPP Ile de France 3  Hôpital Tarnier, 89, rue d’Assas  PARIS, 75006  FRANCE | |
| 1296 | CPP Ile de France 3  Hôpital Tarnier, 89, rue d’Assas  PARIS, 75006  FRANCE | |
| 1309 | CPP Ile de France 3  Hôpital Tarnier Cochin, 89, rue d’Assas  PARIS, 75006  FRANCE | |
| 1317 | CPP Ile de France 3  Hôpital Tarnier Cochin, 89, rue d’Assas  PARIS, 75006  FRANCE | |
| 1318 | CPP Ile de France 3  Hôpital Tarnier, 89, rue d’Assas  PARIS, 75006  FRANCE | |
| **GERMANY** | | |
| 1041 | Ethikkommission der Med. Fakultät der LMU München  Pettenkoferstr. 8a, Prof. Dr. Wolfgang Eisenmenger  80336 Munchen  GERMANY | |
| 1042 | Ethikkommission der Med. Fakultät der LMU München  Pettenkoferstr. 8a, Prof. Dr. Wolfgang Eisenmenger  80336 Munchen  GERMANY | |
| 1045 | Ethikkommission der Med. Fakultät der LMU München  Pettenkoferstr. 8a, Prof. Dr. Wolfgang Eisenmenger  80336 Munchen  GERMANY | |
| 1046 | Ethikkommission der Med. Fakultät der LMU München  Pettenkoferstr. 8a, Prof. Dr. Wolfgang Eisenmenger  80336 Munchen  GERMANY | |
| 1119 | Ethikkommission der Med. Fakultät der LMU München  Pettenkoferstr. 8a, Prof. Dr. Wolfgang Eisenmenger  80336 Munchen  GERMANY | |
| 1135 | Ethikkommission der Med. Fakultät der LMU München  Pettenkoferstr. 8a, Prof. Dr. Wolfgang Eisenmenger  80336 Munchen  GERMANY | |
| 1136 | Aerztekammer Nordrhein  Tersteegenstr. 31  Duesseldorf, 40474  GERMANY | |
|  | Ethikkommission der Med. Fakultät der LMU München  Pettenkoferstr. 8a, Prof. Dr. Wolfgang Eisenmenger  80336 Munchen  GERMANY | |
| 1137 | Ethikkommission der Med. Fakultät der LMU München  Pettenkoferstr. 8a, Prof. Dr. Wolfgang Eisenmenger  80336 Munchen  GERMANY | |
| 1139 | Ethikkommission der Med. Fakultät der LMU München  Pettenkoferstr. 8a, Prof. Dr. Wolfgang Eisenmenger  80336 Munchen  GERMANY | |
| 1184 | Ethikkommission der Med. Fakultät der LMU München  Pettenkoferstr. 8a, Prof. Dr. Wolfgang Eisenmenger  80336 Munchen  GERMANY | |
| 1194 | Ethikkommission der Med. Fakultät der LMU München  Pettenkoferstr. 8 IV, Zi. 1 4.07 bis I 4.10 (Hausanschrift), Postanschrift: Pettenkoferstr. 8a  Munich, 80336  GERMANY | |
| 1207 | Ethikkommission der Med. Fakultät der LMU München  Pettenkoferstr. 8a, Prof. Dr. Wolfgang Eisenmenger  80336 Munchen  GERMANY | |
| 1209 | Ethikkommission der Med. Fakultät der LMU München  Pettenkoferstr. 8a, Prof. Dr. Wolfgang Eisenmenger  80336 Munchen  GERMANY | |
| 1210 | Ethikkommission der Med. Fakultät der LMU München  Pettenkoferstr. 8a, Prof. Dr. Wolfgang Eisenmenger  80336 Munchen  GERMANY | |
| 1211 | Ethikkommission de Landesarztekammer Hessen  Im Vogelsgesang 3  Frankfurt, 60488  GERMANY | |
| 1229 | Ethikkommission der Med. Fakultät der LMU München  Ethikkommission der Med. Fakultät der LMU München, Prof. Dr. Wolfgang Eisenmenger Pettenkoferstr. 8a  Munich, BAVARIA 80336  GERMANY | |
| 1302 | Ethikkommission der Med. Fakultät der LMU München  Pettenkoferstr. 8a, Prof. Dr. Wolfgang Eisenmenger  80336 Munchen  GERMANY | |
| 1303 | Ethikkommission der Med. Fakultaet der LMU Muenchen  Pettenkoferstr. 8a, Prof. Dr. Wolfgang Eisenmenger  Munich, 80336  GERMANY | |
| 1312 | Ethikkommission der Med. Fakultät der LMU München  Pettenkoferstr. 8a, Prof. Dr. Wolfgang Eisenmenger  80336 Munchen  GERMANY | |
| **HUNGARY** | | |
| 1061 | Egeszsegugyi Tudomanyos Tanacs  Zrinyi u.3  Budapest, 1051  HUNGARY | |
| 1100 | Egeszsegugyi Tudomanyos Tanacs  Zrinyi u.3  Budapest, 1051  HUNGARY | |
| 1145 | Egeszsegugyi Tudomanyos Tanacs Klinikai  Arany Janos u. 6-8., Farmakologiai Etikai Bizottsag  Budapest, 1051  HUNGARY | |
| 1227 | Egeszsegugyi Tudomanyos Tanacs  Klinikai Farmakologiai Etikai Bizottsaga  Arany Janos u. 6-8.  Budapest, 1051  HUNGARY | |
| 1228 | Egeszsegugyi Tudomanyos Tanacs  Klinikai Farmakologiai Etikai Bizottsaga  Arany Janos u. 6-8.  Budapest, 1051  HUNGARY | |
| 1284 | Egeszsegugyi Tudomanyos Tanacs  Zrinyi u.3  Budapest, 1051  HUNGARY | |
| **IRELAND** | | |
| 1068 | Clinical Research Ethics Committee of the Cork Teaching Hospitals  6 Little Hanover Street, Lancaster Hall  Cork  IRELAND | |
| 1069 | Clinical Research Ethics Committee of the Cork Teaching Hospitals  6 Little Hanover Street, Lancaster Hall  Cork  IRELAND | |
|  | Mater Multi-Centre Clinical Trials Advisory Group  Eccles Street, Mater Misericordiae University Hospital  Dublin 7  IRELAND | |
| 1098 | Clinical Research Ethics Committee of the Cork Teaching Hospitals  6 Little Hanover Street, Lancaster Hall  Cork  IRELAND | |
|  | Mater Multi-Centre Clinical Trials Advisory Group  Eccles Street, Mater Private Hospital  Dublin 7  IRELAND | |
| 1101 | Clinical Research Ethics Committee of the Cork Teaching Hospitals  6 Little Hanover Street, Lancaster Hall  Cork  IRELAND | |
| 1102 | Clinical Research Ethics Committee of the Cork Teaching Hospitals  6 Little Hanover Street, Lancaster Hall  Cork  IRELAND | |
| 1104 | Clinical Research Ethics Committee of the Cork Teaching Hospitals  6 Little Hanover Street, Lancaster Hall  Cork  IRELAND | |
| 1141 | The Clinical Research Ethics Committee of the Cork Teaching Hospitals  6 Little Hanover Street, Lancaster Hall  Cork  IRELAND | |
| 1247 | Clinical Research Ethics Committee of the Cork Teaching Hospitals  6 Little Hanover Street, Lancaster Hall  Cork  IRELAND | |
| 1248 | Clinical Research Ethics Committee of the Cork Teaching Hospitals  6 Little Hanover Street, Lancaster Hall  Cork  IRELAND | |
| **ITALY** | |  |
| 1050 | Comitato Etico dell'Azienda USL Frosinone  Via Armando Fabi  Frosinone, 03100  ITALY |  |
| 1060 | Comitato Etico  Via Ripamonti, 435, Istituto Europeo Oncologico  Milano, 20141  ITALY |  |
| 1120 | Ethics Committee of the Area Vasta Nord Ovest Autonomous  Section of the Regional Ethic Committee for Clinical Investigation  Via Roma 67  Pisa, 56126  ITALY |  |
|  | Comitato Etico  Via Elio Chianesi, 53  Roma, 00144  ITALY |  |
| 1189 | CEROM (Comitato Etico della Romagna)  Via Moroncelli  Meldola, FORLI 47014  ITALY |  |
| 1191 | Comitato Etico Campania Nord c/o A.O Azienda Ospedaliera San Giuseppe Moscati di Avellino  Contrada Amoretta Citta' Ospedaliera  Avellino, 83100  ITALY |  |
| 1264 | Comitato Etico  Via Elio Chianesi, 53  Roma, 00128  ITALY |  |
| 1265 | Comitato Etico Indipendente  Universitaria di Bologna Policlinico S. Orsola Malpighi  dell'Azienda Ospedaliero, Via Albertoni, 15  Bologna, BO 40138  ITALY |  |
| 1298 | Comitato Etico Per Le Attivita' Biomediche "Carlo Romano"  via Sergio Pansini 5Comitato Etico Per Le Attivita' Biomediche "Carlo Romano", Universita' Degli Studi Di Napoli "Federico II"  Naples, 80131  ITALY |  |
| **JAPAN** | |  |
| 1323 | Aichi Cancer Center Hospital IRB  Kanokoden, Chikusa-ku, 1-1  Nagoya, AICHI 464-8681  JAPAN |  |
| 1324 | National Cancer Center Hospital IRB  Tsukiji, Chuo-ku,, 5-1-1  Tokyo, 104-0045  JAPAN |  |
| 1325 | National Cancer Center Hospital IRB  5-1-1 Tsukiji, Chuo-ku  Tokyo, 104-0045  JAPAN |  |
| 1326 | National Hospital Organization Osaka National Hospital IRB  Hoenzaka, Chuo-ku, 2-1-14  Osaka, OSAKA 540-0006  JAPAN |  |
| 1327 | Hakuaikai Medical Corporation Sagara Hospital Institutional Review Board  3-31, Matsubara-cho  Kagoshima, KAGOSHIMA 892-0833  JAPAN |  |
| 1328 | Iwate Medical University IRB  19-1, Uchimaru  Morioka-shi, IWATE 020-8505  JAPAN |  |
| 1329 | National Hospital Organization Shikoku Cancer Center IRB  160, Kou Minamiumemoto-machi  Matsuyama, EHIME 791-0280  JAPAN |  |
| 1330 | National Hospital Organization Kyushu Cancer Center IRB  3-1-1,Notame, Minami-ku  Fukuoka, FUKUOKA 811-1395  JAPAN |  |
| 1331 | National Hospital Organization Hokkaido Cancer Center Institutional Review Board  Kikusui, Shiroishi-ku, 4-2-3-54  Sapporo-city, HOKKAIDO 003-0804  JAPAN |  |
| 1332 | Chiba Cancer Center IRB  Nitona-cho, Chuo-ku, 666-2  Chiba, CHIBA 260-8717  JAPAN |  |
| 1333 | Hiroshima City Hiroshima Citizens Hospital Institutional Review Board  Moto-machi, Naka-ku, 7-33  Hiroshima, HIROSHIMA 730-8518  JAPAN |  |
| 1334 | Kumamoto University Hospital IRB  1-1-1, Honjo, Chuo-ku  Kumamoto-city, KUMAMOTO 860-8556  JAPAN |  |
| 1335 | Saitama Cancer Center IRB  Komuro, Ina-cho, 780  Kita-adachi-gun, SAITAMA 362-0806  JAPAN |  |
| 1337 | Kumamoto City Hospital IRB  1-1-60 Koto, Higashi-ku  Kumamoto-city, KUMAMOTO 862-8505  JAPAN |  |
| 1340 | Niigata Cancer Center Hospital Institutional Review Board  2-15-3 Kawagishi-cho, Chuo-Ku  Niigata, 951-8566  JAPAN |  |
| KOREA, REPUBLIC OF | |  |
| 1172 | Samsung Medical Center IRB  Samsung Medical Center  81 Irwon-ro, Gangnam-gu  Seoul, 06351  KOREA, REPUBLIC OF |  |
| 1173 | Seoul National University Hospital Institutional Review Board  101, Daehak-ro, Jongno-gu, Seoul National University Hospital Institutional Review Board  Seoul, 03080  KOREA, REPUBLIC OF |  |
| 1174 | Asan Medical Center IRB  Asan Medical Center  88, Olympic-ro, 43-Gil, Songpa-Gu  Seoul, 05505  KOREA, REPUBLIC OF |  |
| 1175 | Seoul National University Bundang Hospital IRB  82, Gumi-ro 173beon-gil, Bundang-gu  Seongnam-si, GYEONGGI-DO 13620  KOREA, REPUBLIC OF |  |
| 1177 | National Cancer Center, Institutional Review Board  323 Ilsan-ro, Ilsandong-gu  Goyang-si, GYEONGGI-DO 10408  KOREA, REPUBLIC OF |  |
| 1179 | Severance Hospital, IRB, Human Research Protection Center  50 Yonsei-ro, Seodaemun-gu, Severance Hospital, Yonsei University Health System  Seoul, 03722  KOREA, REPUBLIC OF |  |
| **NEW ZEALAND** | |  |
| 1169 | Health and Disability Ethics Committee (HDEC)  No 1 The Terrace, Ministry of Health  Wellington, PO Box 5013  NEW ZEALAND |  |
| **NO SCA COUNTRY SPECIFIED** | |  |
| 1103 | Clinical Research Ethics Committee of the Cork Teaching Hospitals  6 Little Hanover Street, Lancaster Hall  Cork  IRELAND |  |
| **POLAND** | |  |
| 1052 | Niezalezna Komisja Bioetyczna ds. Badan Naukowych przy Gdanskim Uniwersytecie Medycznym (GUMed),  M. Sklodowskiej - Curie 3a, Str  Gdansk, 80-210  POLAND |  |
| 1053 | Niezalezna Komisja Bioetyczna do Spraw Badan Naukowych przy GUMed  M. Sklodowskiej Curie 3A, Str.  Gdansk, 80-211  POLAND |  |
| 1099 | Niezalezna Komisja Bioetyczna do Spraw Badan Naukowych przy GUMed  M. Sklodowskiej - Curie 3a  Gdansk, 80-211  POLAND |  |
| 1245 | Niezalezna Komisja Bioetyczna do Spraw Badan Naukowych przy GUMed  M. Sklodowskiej - Curie 3a  Gdansk, 80-211  POLAND |  |
| RUSSIAN FEDERATION | |  |
| 1051 | Local Ethics Committee  Kursk Regional Clinical Oncology Dispensary  20, ul Pirogova, Healthcare Committee of Kursk Region  Kursk, 305035  RUSSIAN FEDERATION |  |
|  | Ethics Council under the Ministry of Health of the Russian Federation  3, Rakhmanovskiy pereulok  Moscow, 127994  RUSSIAN FEDERATION |  |
| 1055 | LEC of State Budget Healtcare Institution  37-39 Liteiny prospect, "Leningrad Region Oncology Dispensary"  Saint-Petersburg, 191014  RUSSIAN FEDERATION |  |
|  | Ethics Council under the Ministry of Health of the Russian Federation  3, Rakhmanovskiy pereulok  Moscow, 127994  RUSSIAN FEDERATION |  |
| 1056 | Ethics Council at the Ministry of Health of the Russian Federation  3, Rakhmanovskiy pereulok  Moscow, 127994  RUSSIAN FEDERATION |  |
|  | LEC of SBMI Republican Clinical Oncology Dispensary under the Ministry of health Care of RB  73/1. Oktyabrya pr.  Ufa, RUSSIA 450054  RUSSIAN FEDERATION |  |
| 1058 | LEC of Saint-Petersburg State Budget Healthcare Institution (SBHCI)  City Clinical Oncology Dispensary  56, Veteranov prospect  Saint-Petersburg, 197022  RUSSIAN FEDERATION |  |
|  | Ethics Council of the Ministry of Health of the Russian Federation  3 Rakhmanovsky pereulok  Moscow, 127994  RUSSIAN FEDERATION |  |
| 1059 | LEC of State Budget institution of Healthcare Omsk Regional Clinical Oncology Dispensary  9/1, Zavertyaeva, Str.  Omsk, 644013  RUSSIAN FEDERATION |  |
|  | Ethics Council of the Ministry of Health of the Russian Federation  3 Rakhmanovsky pereulok  Moscow, 127994  RUSSIAN FEDERATION |  |
| 1124 | Local Ethical Committee  State Medicoprophylactic Institution 'Chelyabinsk Regional Oncology Center'  42, Blukhera str.  Chelyabinsk, 454076  RUSSIAN FEDERATION |  |
|  | Ethics Council of the Ministry of Health of the Russian Federation  3 Rakhmanovsky pereulok  Moscow, 127994  RUSSIAN FEDERATION |  |
| 1216 | Ethics Committee of N.N.Blokhin Cancer Research Center RAMS  N.N.Blokhin Cancer Research Center RAMS  24, Kashirskoye sh.  Moscow, 115478  RUSSIAN FEDERATION |  |
|  | Ethics Council of the Ministry of Health of the Russian Federation  3 Rakhmanovsky pereulok  Moscow, 127994  RUSSIAN FEDERATION |  |
| 1286 | LEC based on Non-State HealthCare agency "Road Clinical Hospital of PLC" Russian Railways"  27, prospect MechnikovaNon-State Institution of Healthcare, RZHD OJSC RR  St. Petersburg, 195271  RUSSIAN FEDERATION |  |
|  | Ethics Council under the Ministry of Health of the Russian Federation  3, Rakhmanovskiy pereulok  Moscow, 127994  RUSSIAN FEDERATION |  |
| 1287 | Expert Board/Ethics Committee of Republic  Clinical Oncology Dispensary of Ministry of Health of Republic of Tatarstan  29, Sibirsky trakt  Kazan, 420029  RUSSIAN FEDERATION |  |
|  | Ethics Council of the Ministry of Health of the Russian Federation  3 Rakhmanovsky pereulok  Moscow, 127994  RUSSIAN FEDERATION |  |
| 1297 | Ethics Committee of State Educational Institution of Higher Professional Education Ryazan State  Medical University I.P. Pavlov, 9, Vysokovoltnaya ulitsa  Ryazan, 390026  RUSSIAN FEDERATION |  |
|  | Ethics Council at the Ministry of Health of the Russian Federation  3, Rakhmanovskij pereulok  Moscow, 127994  RUSSIAN FEDERATION |  |
| 1308 | EC of SAHI of Moscow "Moscow City Oncology Hospital #62 of Health Department of Moscow"  Moscow Healthcare Department  Settlement Istra 27 bld 1-26, Krasnogorskiy Disctrict  Moscow area, 143423  RUSSIAN FEDERATION |  |
|  | Ethics Council of the Ministry of Health of the Russian Federation  3 Rakhmanovsky pereulok  Moscow, 127994  RUSSIAN FEDERATION |  |
| **SPAIN** | |  |
| 1079 | Comite etico de investigacion clinica  C/Feixa Llarga, s/nHospital universitario de Bellvitge, L'Hospitalet del Llobregat, secretaria administrativa del CEIC. Edifici de recerca  Barcelona, 08907  SPAIN |  |
| 1080 | Comite Etico de Investigación Clínica. Hospital Universitari de Bellvitge  Secretaría Administrativa del CEIC,Edifici Unitat de Recerca.  L'Hospitalet de Llobregat, C/ Feixa Llarga, s/n  Barcelona, 08907  SPAIN |  |
| 1081 | Comite Etico de Investigacion Clinica, Hospital Universitario de Bellvitge  Secretaria Administrativa del CEIC, Edifici Unitat de Recerca  C/ Feixa Llarga, s/n  L'Hospitalet de Llobregat, BARCELONA 08907  SPAIN |  |
| 1082 | Comite Etico de Investigacion Clinica, Hospital Universitario de Bellvitge  Secretaria Administrativa del CEIC, Edifici Unitat de Recerca  C/ Feixa Llarga, s/n  L'Hospitalet de Llobregat, BARCELONA 08907  SPAIN |  |
|  | Comite Etico de Investigacion Clinica de Las Islas Baleares  Camino de Jesus, 38, Conselleria de Salut i Consum  Palma de Mallorca, ISLAS BALEARES 07011  SPAIN |  |
| 1083 | Comite Etico de Investigación Clínica. Hospital Universitari de Bellvitge  Secretaría Administrativa del CEIC,Edifici Unitat de Recerca.  L'Hospitalet de Llobregat, C/ Feixa Llarga, s/n  Barcelona, 08907  SPAIN |  |
| 1084 | Comite Etico de Investigacion Clinica, Hospital Universitario de Bellvitge  Secretaria Administrativa del CEIC, Edifici Unitat de Recerca  C/ Feixa Llarga, s/n  L'Hospitalet de Llobregat, BARCELONA 08907  SPAIN |  |
| 1085 | Comite Etico de Investigacion Clinica, Hospital Universitario de Bellvitge  Secretaria Administrativa del CEIC, Edifici Unitat de Recerca  C/ Feixa Llarga, s/n  L'Hospitalet de Llobregat, BARCELONA 08907  SPAIN |  |
| 1086 | Comite Etico de Investigacion Clinica, Hospital Universitario de Bellvitge  Secretaria Administrativa del CEIC, Edifici Unitat de Recerca  C/ Feixa Llarga, s/n  L'Hospitalet de Llobregat, BARCELONA 08907  SPAIN |  |
| 1087 | Comite Etico de Investigacion Clinica, Hospital Universitario de Bellvitge  Secretaria Administrativa del CEIC, Edifici Unitat de Recerca  C/ Feixa Llarga, s/n  L'Hospitalet de Llobregat, BARCELONA 08907  SPAIN |  |
| 1088 | Comite etico de investigacion clinica  C/Feixa Llarga, s/nHospital universitario de Bellvitge, L'Hospitalet del Llobregat, secretaria administrativa del CEIC. Edifici de recerca  Barcelona, 08907  SPAIN |  |
| 1089 | Comite Etico de Investigacion Clinica, Hospital Universitario de Bellvitge  Secretaria Administrativa del CEIC, Edifici Unitat de Recerca  C/ Feixa Llarga, s/n  L'Hospitalet de Llobregat, BARCELONA 08907  SPAIN |  |
| 1090 | Comite etico de investigacion clinica  Edifici Unitat de recerca  secretaria administrativa del CEIC.C/Feixa Llarga, s/n, Hospital universitario de Bellvitge L'Hospitalet del Llobregat  Barcelona, 08907  SPAIN |  |
| 1091 | Comite etico de investigacion clinica  C/Feixa Llarga, s/nHospital universitario de Bellvitge, L'Hospitalet del Llobregat, secretaria administrativa del CEIC. Edifici de recerca  Barcelona, 08907  SPAIN |  |
| 1092 | Comite Etico de Investigacion Clinica  Secretaria Administrativa del CEIC, Edifici Unitat de Recerca  Hospital Universitario de Bellvitge, C/ Feixa Llarga, s/n  L'Hospitalet de Llobregat, BARCELONA 08907  SPAIN |  |
| 1105 | Comite Etico de Investigacion Clinica  Secretaría Administrativa del CEIC, Edifici Unitat de Recerca.  Hospital Universitario de Bellvitge, L'Hospitalet de Llobregat, C/Feixa Llarga, s/n  Barcelona, 08907  SPAIN |  |
| 1106 | Comite Etico de Investigacion Clinica  Secretaría Administrativa del CEIC, Edifici Unitat de Recerca.  Hospital Universitario de Bellvitge, L'Hospitalet de Llobregat, C/Feixa Llarga, s/n  Barcelona, 08907  SPAIN |  |
| 1107 | Comite Etico de Investigacion Clinica  Secretaría Administrativa del CEIC, Edifici Unitat de Recerca.  Hospital Universitario de Bellvitge, L'Hospitalet de Llobregat, C/Feixa Llarga, s/n  Barcelona, 08907  SPAIN |  |
| 1116 | Comite Etico de Investigacion Clinica  Secretaría Administrativa del CEIC, Edifici Unitat de Recerca.  Hospital Universitario de Bellvitge, L'Hospitalet de Llobregat, C/Feixa Llarga, s/n  Barcelona, 08907  SPAIN |  |
| 1117 | Comite Etico de Investigacion Clinica  Secretaría Administrativa del CEIC, Edifici Unitat de Recerca.  Hospital Universitario de Bellvitge, L'Hospitalet de Llobregat, C/Feixa Llarga, s/n  Barcelona, 08907  SPAIN |  |
| 1146 | Hospital Fundación de Alcorcón  C/ Budapest 1 - Planta SótanoComité Ético de Investigación Clínica - Área 8, Fundación Hospital Alcorcón  Madrid, ALCORCÓN 28922  SPAIN |  |
|  | Comite etico de investigacion clinica  C/Feixa Llarga, s/nHospital universitario de Bellvitge, L'Hospitalet del Llobregat, secretaria administrativa del CEIC. Edifici de recerca  Barcelona, 08907  SPAIN |  |
| 1147 | Comite etico de investigacion clinica  C/Feixa Llarga, s/nHospital universitario de Bellvitge, L'Hospitalet de Llobregat, secretaria administrativa del CEIC. Edifici de recerca  Barcelona, 08907  SPAIN |  |
| 1149 | Comite Etico de Investigacion Clinica  Secretaría Administrativa del CEIC, Edifici Unitat de Recerca.  Hospital Universitario de Bellvitge, L'Hospitalet de Llobregat, C/Feixa Llarga, s/n  Barcelona, 08907  SPAIN |  |
| **TAIWAN** | |  |
| 1260 | Institutional Review Board, Koo Foundation Sun Yat-Sen Cancer Center  No.125, Lih-Der Road, Taiwan R.O.C  Bei-Tou District, TAIPEI 11259  TAIWAN |  |
| 1261 | Institutional Review Board, Taipei Veterans General Hospital  No.201, Sec. 2, Shipai Rd, Beitou District  Taipei, 11217  TAIWAN |  |
| 1266 | Mackay Memory Hospital Institutional Review Board  No. 92, Section 2, Zhongshan North RoadMACKAY MEMORIAL HOSPITAL Institutional Review Board, Zhongshan District, 8 F  Taipei, 10449  TAIWAN |  |
| 1311 | National Taiwan University Hospital, Research Ethics Committee  Changde St., Zhongzheng Dist., No.1  Taipei City, 10002  TAIWAN |  |
| **UKRAINE** | |  |
| 1143 | Ethics Committee of the MI  31, Blyzhnya Str., Dnipropetrovsk City Multidisciplinary Clinical Hospital No. 4 of the Dnipropetrovsk City Council  Dnipro, 49102  UKRAINE |  |
| 1185 | Local Ethic Committee of Lviv State  Oncologic Regional Treatment and Diagnostic Center  2A, Ja. Gashek St.  Lviv, 79031  UKRAINE |  |
| 1186 | Local Committee of Ethic Issues of Municipal Institution  Local Committee of Ethic Issues of Municipal Institution Zaporizhzhia Regional Clinical Oncology  177a, Kultuma Str., Dispensary" ZRA  Zaporizhzhia, 69040  UKRAINE |  |
| 1295 | Local Ethic Committee of Uzhgorod of Central Municipal Clinical Hospital  20, Hryboyedova Str.  Uzhgorod, N/A 88000  UKRAINE |  |
| 1300 | Local Ethic Committee of MMI "Makiivka City Hospital No.2  of Donetsk Region"  12 Ferhanska str  Makiivka, N/A 86120  UKRAINE |  |
| 1301 | Commission of Ethic Issues of Municipal Non-Profit Enterprise "Regional Centre of Oncology"  4, Lisoparkivs'ka Str.  Kharkiv, 61070  UKRAINE |  |
| 1304 | Local Committee of Ethic Issues of Regional Municipal Establishment  Sumy Regional Clinical Oncology Dispensary  31, Pryvokzalna Str.  Sumy, 40005  UKRAINE |  |
| **UNITED KINGDOM** | |  |
| 1171 | South East Coast-Brighton and Sussex REC  80 London Road, Ground Floor, Skipton House  London, SE1 6LH  UNITED KINGDOM |  |
| 1249 | South East Coast- Brighton and Sussex REC  80 London Road Ground Floor, Skipton house  London, SE1 6LH  UNITED KINGDOM |  |
| 1250 | NRES Committee south East Coast-Brighton and Sussex health Research Authority  80 London Road, Ground Floor Skipton House  London, SE1 6LH  UNITED KINGDOM |  |
| 1251 | South East Coast- Brighton and Sussex REC  80 London Road Ground Floor, Skipton house  London, SE1 6LH  UNITED KINGDOM |  |
| 1271 | NRES Committee London - Central  80 London RoadNRES Committee South East Coast - Brighton and Sussex Health Research Authority, Ground Floor, Skipton House  London, SE1 6LH  UNITED KINGDOM |  |
| 1310 | South East Coast- Brighton and Sussex REC  80 London Road Ground Floor, Skipton house  London, SE1 6LH  UNITED KINGDOM |  |
| 1320 | South East Coast- Brighton and Sussex REC  80 London Road Ground Floor, Skipton house  London, SE1 6LH  UNITED KINGDOM |  |
| **UNITED STATES** | |  |
| 1001 | Western Institutional Review Board  1019 39th Ave SE, Ste 120  Puyallup, WA 98374-2115  UNITED STATES |  |
| 1003 | Western Institutional Review Board  1019 39th Ave SE, Ste 120  Puyallup, WA 98374-2115  UNITED STATES |  |
| 1004 | Sparrow Institution Research Review Committee Office of Research Oversight and Compliance  Sparrow Institutional Review Board, 1215 E Michigan Ave. PO Box 30480  Lansing, MI 48090-7890  UNITED STATES |  |
| 1005 | Western Institutional Review Board  1019 39th Ave SE, Ste 120  Puyallup, WA 98374-2115  UNITED STATES |  |
| 1006 | Western Institutional Review Board  1019 39th Ave SE, Ste 120  Puyallup, WA 98374-2115  UNITED STATES |  |
| 1007 | Western Institutional Review Board  1019 39th Ave SE, Ste 120  Puyallup, WA 98374-2115  UNITED STATES |  |
| 1008 | Western Institutional Review Board  1019 39th Ave SE, Ste 120  Puyallup, WA 98374-2115  UNITED STATES |  |
| 1009 | UCLA - Office of the Human Research Protection Program  10889 Wilshire Blvd., Ste. 830  Los Angeles, CA 90024  UNITED STATES |  |
| 1010 | Western Institutional Review Board  1019 39th Ave SE, Ste 120  Puyallup, WA 98374-2115  UNITED STATES |  |
| 1011 | OHSU Institutional Review Board  3181 Sw Sam Jackson Park Rd, L106-RI  Portland, OR 97239  UNITED STATES |  |
| 1012 | Western Institutional Review Board  1019 39th Ave SE, Ste 120  Puyallup, WA 98374-2115  UNITED STATES |  |
| 1013 | Western Institutional Review Board  1019 39th Ave SE, Ste 120  Puyallup, WA 98374-2115  UNITED STATES |  |
| 1014 | Western Institutional Review Board  1019 39th Ave SE, Ste 120  Puyallup, WA 98374-2115  UNITED STATES |  |
| 1017 | US Oncology Incorporated  10101 Woodloch Forest Dr, Institutional Review Board  The Woodlands, TX 77380  UNITED STATES |  |
| 1019 | Kaiser Permanente Northwest Institutional Review Board  3800 N Interstate Ave, Research Subjects Protection Ofc  Portland, OR 97227  UNITED STATES |  |
| 1020 | Western Institutional Review Board  1019 39TH AVE SE  PUYALLUP, WA 98374  UNITED STATES |  |
| 1023 | University of Miami Institutional Review Board  1400 NW 10th Ave, Ste 1200A  Miami, FL 33136  UNITED STATES |  |
| 1024 | Western Institutional Review Board  1019 39th Ave SE, Ste 120  Puyallup, WA 98374-2115  UNITED STATES |  |
| 1025 | Western Institutional Review Board  1019 39th Ave SE, Ste 120  Puyallup, WA 98374-2115  UNITED STATES |  |
| 1026 | Columbia University Medical Center Institutional Review Board  154 Haven Ave, First Fl  New York, NY 10032  UNITED STATES |  |
| 1028 | Human Subject Protection Program Office (HSPPO)  MedCenter One  501 E Broadway, Ste 200  Louisville, KY 40202  UNITED STATES |  |
| 1029 | Western Institutional Review Board  1019 39th Ave SE, Ste 120  Puyallup, WA 98374-2115  UNITED STATES |  |
| 1030 | West Virginia University Institutional Review Board  Chestnut Ridge Research Bldg, 866 Chestnut Ridge Rd, PO Box 6845  Morgantown, WV 26506  UNITED STATES |  |
| 1031 | University of Southern California Health Science Campus Institutional Review Board  LAC/USC Medical Center  1200 N State St, General Hospital Ste 4700  Los Angeles, CA 90033  UNITED STATES |  |
| 1032 | Western Institutional Review Board  1019 39th Ave SE, Ste 120  Puyallup, WA 98374-2115  UNITED STATES |  |
| 1033 | Western Institutional Review Board  1019 39th Ave SE, Ste 120  Puyallup, WA 98374-2115  UNITED STATES |  |
| 1035 | Mercy Hospital St. Louis Institutional Review Board  621 S. New Ballas Rd., Ste 6002B  St. Louis, MO 63141  UNITED STATES |  |
| 1040 | Western Institutional Review Board  1019 39th Ave SE, Ste 120  Puyallup, WA 98374-2115  UNITED STATES |  |
| 1078 | UCSF IRB  3333 California St, Ste 315  San Francisco, CA 94118  UNITED STATES |  |
| 1109 | US Oncology Incorporated  10101 Woodloch Forest Dr, Institutional Review Board  The Woodlands, TX 77380  UNITED STATES |  |
| 1110 | US Oncology Incorporated  10101 Woodloch Forest Dr, Institutional Review Board  The Woodlands, TX 77380  UNITED STATES |  |
| 1111 | Chesapeake IRB  6940 Columbia Gateway Dr  Columbia, MD 21046-3403  UNITED STATES |  |
| 1112 | US Oncology Incorporated  10101 Woodloch Forest Dr, Institutional Review Board  The Woodlands, TX 77380  UNITED STATES |  |
| 1125 | US Oncology Incorporated  10101 Woodloch Forest Dr, Institutional Review Board  The Woodlands, TX 77380  UNITED STATES |  |
| 1126 | US Oncology Incorporated  10101 Woodloch Forest Dr, Institutional Review Board  The Woodlands, TX 77380  UNITED STATES |  |
| 1127 | Western Institutional Review Board  1019 39th Ave SE, Ste 120  Puyallup, WA 98374-2115  UNITED STATES |  |
| 1128 | US Oncology Incorporated  10101 Woodloch Forest Dr, Institutional Review Board  The Woodlands, TX 77380  UNITED STATES |  |
| 1129 | Yale University Institutional Review Board #2, 3, 4B, 5  150 Munson StreetHuman Investigation Committee I, II, III, IV, 3rd Fl  New Haven, CT 06520  UNITED STATES |  |
| 1131 | Western Institutional Review Board  1019 39th Ave SE, Ste 120  Puyallup, WA 98374-2115  UNITED STATES |  |
| 1132 | US Oncology Incorporated  10101 Woodloch Forest Dr, Institutional Review Board  The Woodlands, TX 77380  UNITED STATES |  |
| 1133 | University of Michigan Review Board for Human Subject Research (IRBMED)  2800 Plymouth Rd  Ann Arbor, MI 48109  UNITED STATES |  |
| 1134 | Department of Research Programs, Walter Reed National Military Medical Center IRB  8901 Wiscosin AvenueBuilding 17, Rm 3055  Bethesda, MD 20889-5600  UNITED STATES |  |
| 1162 | UT MD Anderson Cancer Center Institutional Review Board  7007 Bertner Ave., Unit 1637  Houston, TX 77030  UNITED STATES |  |
| 1198 | Western Institutional Review Board  1019 39th Ave SE, Ste 120  Puyallup, WA 98374-2115  UNITED STATES |  |
| 1201 | Human Research Protections Office  UMB BioPark OneUniversity of Maryland School of Medicine, 800 W Baltimore St, Ste 100  Baltimore, MD 21201  UNITED STATES |  |
| 1203 | US Oncology Incorporated  10101 Woodloch Forest Dr, Institutional Review Board  The Woodlands, TX 77380  UNITED STATES |  |
| 1213 | HealthPartners Institute Institutional Review Board  3311 E. Old Shakopee Rd.  Bloomington, MN 55425  UNITED STATES |  |
| 1214 | Western Institutional Review Board  1019 39th Ave SE, Ste 120  Puyallup, WA 98374-2115  UNITED STATES |  |
| 1230 | Western Institutional Review Board  1019 39th Ave SE, Ste 120  Puyallup, WA 98374-2115  UNITED STATES |  |
| 1235 | Kaiser Permanente Southern California Institutional Review Board  393 E Walnut St, 2nd Fl  Pasadena, CA 91188  UNITED STATES |  |
| 1246 | Western Institutional Review Board  1019 39th Ave SE, Ste 120  Puyallup, WA 98374-2115  UNITED STATES |  |
| 1280 | Western Institutional Review Board  1019 39th Ave SE, Ste 120  Puyallup, WA 98374-2115  UNITED STATES |  |
| 1281 | Norwalk Hospital  34 Maple St, Institutional Review Board  Norwalk, CT 06856  UNITED STATES |  |
| 1315 | US Oncology Incorporated  10101 Woodloch Forest Dr, Institutional Review Board  The Woodlands, TX 77380  UNITED STATES |  |
| 1316 | MedStar Health Research Institute  Sw 104 Medical Dental Bldg, 3900 Reservoir Rd, Nw, Georgetown University Oncology Review Board  Washington, DC 20057-2197  UNITED STATES |  |
| 1350 | Stanford Research Compliance Office  1501 S. California Ave.  Palo Alto, CA 94304  UNITED STATES |  |
| 1358 | Western Institutional Review Board  1019 39th Ave SE, Ste 120  Puyallup, WA 98374-2115  UNITED STATES |  |
| 1359 | Western Institutional Review Board  1019 39th Ave SE, Ste 120  Puyallup, WA 98374-2115  UNITED STATES |  |
| 1362 | Western Institutional Review Board  1019 39th Ave SE, Ste 120  Puyallup, WA 98374-2115  UNITED STATES |  |
| 1364 | Western Institutional Review Board  1019 39 th Ave Se, Ste 120  Puyallup, WA 98374  UNITED STATES |  |
| 1365 | Western Institutional Review Board  1019 39 th Ave Se, Ste 120  Puyallup, WA 98374  UNITED STATES |  |
| 1372 | Western Institutional Review Board  1019 39th Ave SE, Ste 120  Puyallup, WA 98374  UNITED STATES |  |
| 1373 | Western Institutional Review Board  1019 39th Ave SE, Ste 120  Puyallup, WA 98374-2115  UNITED STATES |  |
| 1378 | Western Institutional Review Board  1019 39th Ave., SE, Ste 120  Puyallup, WA 98374  UNITED STATES |  |
| 1387 | Western Institutional Review Board  1019 39 th Ave Se, Ste 120  Puyallup, WA 98374  UNITED STATES |  |
